# Analysis of the Pediatric Radiotherapy Landscape in Mexico and a Subsequent Educational e-Contouring Intervention

**DOI:** 10.1200/GO.22.00372

**Published:** 2023-06-29

**Authors:** Raymond B. Mailhot Vega, Beatriz E. Garcia Robles, Christopher G. Morris, Kara Buss, Ulises Mejia, Adela Poitevin, Maria Fatima Chilaca Rosas, Heynar Perez Villanueva, Jesus Armando Felix Leyva, Daniel J. Indelicato, Dolores De la Mata

**Affiliations:** ^1^ Department of Radiation Oncology, University of Florida College of Medicine, Jacksonville, FL; ^2^ Hospital Infantil Teleton de Oncología, Santiago de Querétaro, México; ^3^ Instituto Nacional de Cancerología, Hospital Infantil de México, Ciudad de México, México; ^4^ Hospital Medica Sur, Ciudad de México, México; ^5^ Hospital de Oncología de Centro Médico Siglo XXI, Ciudad de México, México; ^6^ Hospital Infantil de México Federico Gómez, Santiago de Querétaro, México; ^7^ Oncología Consultorio, León, México; ^8^ Centro Médico ABC, Ciudad de México, México

## Abstract

**PURPOSE:**

Mexico and Central America have the highest childhood cancer incidence in the West. Pediatric-specific oncology knowledge contributes to the disparity. We sought to (1) determine the self-identified treatment patterns and needs of Mexican pediatric radiation oncologists and (2) pilot a workshop to improve contouring accuracy.

**MATERIALS AND METHODS:**

Partnering with local experts and the Sociedad Mexicana de Radioterapeutas (SOMERA), a 35-question survey was designed to ascertain pediatric radiotherapy capacity and distributed through the SOMERA listserv. The most challenging malignancies were selected for workshop. Participants received precontouring and postcontouring homework to assess improvement per the Dice metric. The Wilcoxon sign-rank test was used for comparative statistics.

**RESULTS:**

Ninety-four radiation oncologists attempted and 79 completed the survey. Forty-four (76%) felt comfortable treating a pediatric patient, and 36 (62%) were familiar with national protocols for pediatric treatment. Most had access to nutrition, rehabilitation, endocrinology, and anesthesia; 14% had access to fertility services and 27% to neurocognitive support; 11% noted no support, and only one respondent had child-life support. The postsurvey contouring workshop was conducted for high-grade glioma, medulloblastoma, and Hodgkin lymphoma. Significant improvements were seen in all target volumes.

**CONCLUSION:**

We present the first national survey of Mexico's pediatric radiotherapy capacity and Latin American e-contouring educational intervention with preworkshop and postworkshop Dice metrics, noting statistically significant improvement in all target volumes. Participation improved compared with prior experience through SOMERA partnership and Continuing Medical Education incentivization.

## Teaser

The first national survey of Mexican pediatric radiotherapy capacity and successful educational intervention.

## INTRODUCTION

The incidence of childhood cancer in Mexico outpaces its incidence in North and South America.^1^ A 2019 Lancet report demonstrated that Mexico and Central America have the highest incidence of childhood cancers in the Western Hemisphere. In fact, cancer is the second most common cause of death for children between four and 15 years of age in Mexico.^2^ In most developed countries, contemporary diagnosis and treatment yield survival rates up to 80%, but in low- and middle-income countries (LMICs) such as Mexico, survival rates vary from 5% to 60%.^2^ For example, in Mexico, the 5-year survival estimates for ependymoma and central nervous system germ cell tumors, two of the most common pediatric malignancies, are approximately 20%, which is dramatically less than the survival outcomes reported in the United States.^3^

**CONTEXT**

**Key Objective**
To determine the pediatric radiotherapy capacity, practice patterns, and challenges faced by Mexican radiation oncologists and implement an educational intervention on the basis of those needs.
**Knowledge Generated**
Mexican radiation oncologists who treat children noted pressure points regarding access to multidisciplinary teams, fertility services, neurocognitive support, child life, and treatment delays. We demonstrated improved contouring in target volumes for those pediatric malignancies respondents treated most commonly.
**Relevance**
This is the first national survey to report Mexican pediatric radiotherapy capacity to provide a cross-sectional landscape analysis with self-identified needs. We demonstrate success with the first published Spanish language e-contouring educational intervention.

A separate Lancet commission tasked with evaluating cancer control in Latin America assessed pediatric oncology services, emphasizing their complex and costly management.^4^ A report evaluating radiotherapy services in Mexico noted significant heterogeneity in the available technology, ranging from two-dimensional (2D) film-based treatment planning to computed tomography–based intensity-modulated radiotherapy.^5^ With an increasing gap in radiation oncology capacity and rising cancer incidence, there is great demand for more modern radiotherapy facilities and equipment as well as properly educated health care professionals.^6,7^ Valsecchi et al^8^ found that lack of expertise was a possible cause of outcome disparities. In describing Mexico's particular environment, the Lancet commission highlighted challenges the public system faces given the insufficient numbers of trained pediatric oncologists and oncology nurses.^4,9^ In fact, a 25%-40% shortage of oncologists was anticipated for 2020 compared with 2005.^4^

As LMICs acquire newer machines and treatment modalities, it is a challenge for practitioners to gain the technical expertise required for proper tumor targeting,^10^ which differs widely between modalities. Retrospective investigations of prospective oncology trials have demonstrated the negative effect that poor radiotherapy design quality and consistency can have on patient outcomes.^11,12^ To that end, e-contouring sessions—workshops where participants contour practice cases and compare their results with those of an expert—are one way in which radiation oncologists gain technical knowhow.^13^ Although the American Society for Radiation Oncology, the Pediatric Radiation Oncology Society (PROS), and European Society for Radiotherapy & Oncology (ESTRO) regularly provide such workshops, Latin American opportunities are more sporadic. A systematic review from 2020 failed to identify any contouring workshops published in Latin America.^14^ Owing to this need, we sought to address a possible root cause of disparities in childhood cancer outcomes through a targeted educational intervention.

## MATERIALS AND METHODS

This project had two primary aims: (1) survey Mexican radiation oncologists to determine their treatment patterns, self-reported needs, and concerns regarding pediatric cases and (2) measure the change in pediatric radiotherapy knowledge before and after a treatment planning assessment.

### Aim One: The Landscape Survey

An international team comprised American and Mexican pediatric radiation oncology experts to devise the questions to include in the survey. Our goal was to identify the pediatric radiotherapy knowledge and needs of practicing radiation oncologists in Mexico through questions pertaining to (1) frequency and types of pediatric cases seen, (2) radiotherapy modalities available, (3) comfort of radiation oncologists in seeing pediatric cases, (4) met and unmet needs of radiation oncologists, and (5) knowledge regarding radiotherapy techniques for pediatric cases. Our stakeholder planning combining US and Mexican pediatric oncology experts and Mexican leaders from the Sociedad Mexicana de Radioterapeutas (SOMERA) began in the summer of 2019 and continued for approximately 6 months to determine the questions and format of the survey.

The survey consisted of 35 questions and was disseminated in partnership with the national SOMERA through their membership listserv and subsequently promoted through social media. The survey was developed on Qualtrics (Qualtrics, Provo, UT). Please refer to the appendix to see a full copy of the survey. The initial e-mail from SOMERA denoted a target date for survey completion, with two reminder e-mails sent. The study was approved under institutional review board 201902197. Approval was obtained at both University of Florida in Jacksonville, FL and ABC Medical Center in Ciudad de México, México.

### Aim Two: Educational Intervention

We also sought to provide pediatric malignancy contour training for radiation oncologists. This aim included three intervention phases: (1) preintervention assessment of knowledge, (2) on-site educational intervention, and (3) postintervention assessment of knowledge. Our plan was to use the survey completed in aim 1 to determine the three malignancies to select for the workshop, specifically the tumors that respondents noted they treated the most. If three malignancies did not clearly stand out, the stakeholders decided they would focus on treatment planning in Hodgkin lymphoma, ependymoma, and medulloblastoma.

Because of the COVID-19 pandemic, the annual in-person meeting for SOMERA was converted to a virtual event. As such, the workshop was scheduled for November 28, 2020, an event cosponsored by the PROS. Attendees were given 2 weeks before and after the workshop to complete preworkshop and postworkshop homework. Contouring was conducted using the web-based platform EduCase (RadOnc eLearning Center Inc, Fremont, CA). Participants contoured assigned homework structures before and after the workshop to assess improvements in accuracy as measured by the Dice metric, a value between 0 and 1, wherein 1 indicates total matching of structure delineation and 0 represents no commonality. We used EduCase to store contouring information and calculate the Dice scores. For Dice score calculation, participant contours were compared against gold standard contours which were reviewed and prepared in consensus by D.J.I., R.B.M.V., and D.D.l.M. As noted in a systematic review of educational interventions, Dice similarity is the most common evaluation metric for educational interventions.^14^ The Dice metric has similarly been used by ESTRO for their educational projects.^15^ Given the established framework of evidence, we similarly selected the Dice metric for quantitative evaluation of contouring. Each case was accompanied by a lecture describing the management of that malignancy. Moreover, a presentation was delivered by a dosimetrist regarding plan evaluation with a focus on craniospinal irradiation. Contouring was performed on the EduCase platform. Homework completion was encouraged by SOMERA's offering Continuing Medical Education (CME) credits.

### Statistical Analyses

For the survey in aim 1, data collection was performed through Qualtrics. Descriptive statistics were conducted given the nature of the responses and included frequencies, averages, medians, and ranges. Comparative statistics between participants were conducted with logistic regression. We used an α of .05 for significance. For the educational contouring intervention, the outcome of interest was the Dice metric as above. Comparative statistics evaluating the efficacy of contouring intervention used the Wilcoxon sign-rank test which is a paired test. Statistical analyses were performed on JMP Pro version 16.1.0 (SAS, Cary, NC).

## RESULTS

### Aim One: The Survey Results

**FIG 1:**
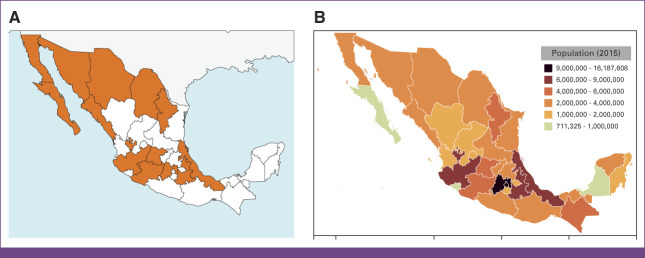
(A) A geographic representation of the states wherein the survey respondents practice. (B) A geographic heatmap displaying the Mexican states by population.

The survey was open from July 11, 2020, through August 30, 2020. The SOMERA listserv includes 350 respondents. 94 (27%) radiation oncologists attempted and 79 (23%) completed the survey, representing 20 different cities across Mexico. The email invitation to the survey specified the survey was related to pediatric radiotherapy capacity. Figure 1 depicts the states where survey respondents practice and the population within those states.

Of the 79 respondents, 58 (73%) they had treated a pediatric patient within the past year with those 58 having a median 9 years of practice (1-38). Furthermore, 44 (76%) respondents felt comfortable with treating a pediatric patient. The median number of pediatric cases seen in the past year by those respondents who had seen a pediatric patient was 9.5 (range, 1-195 cases). Comfort in treating a pediatric patient was not significantly associated with time in practice (*P* = .11) nor average number of pediatric patients seen in the past year (*P* = .42). Thirty-six respondents (62%) said they were familiar with Mexican national protocols for pediatric cancer treatment and 95% noted that they learned pediatric management through conferences. The three most common tumors treated were Hodgkin lymphoma (median, 2; range, 0-21), medulloblastoma (median, 1.5; range, 0-20), and Wilms tumor (median, 1; range, 0-20), respectively (Table 1). The median percentage of pediatric patients treated palliatively was 10% (range, 0%-100%). Twenty-six percent reported access to a multidisciplinary tumor board.

**TABLE 1:**
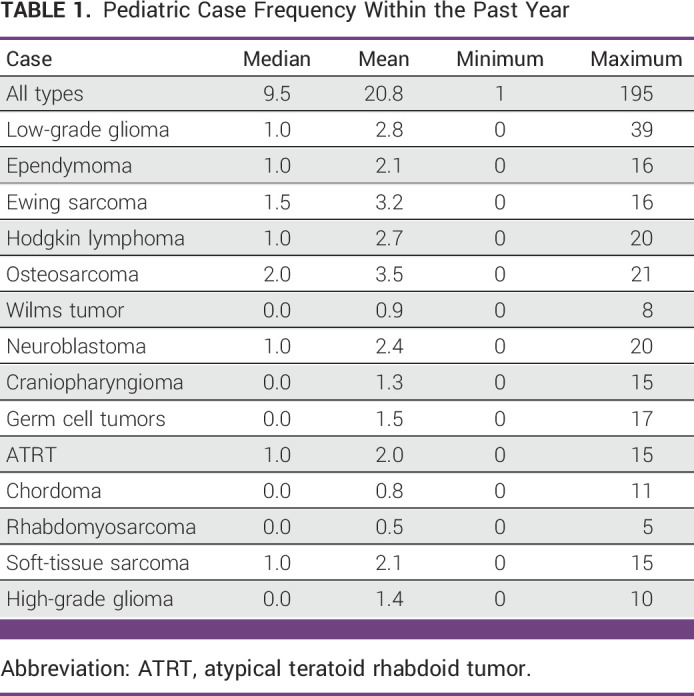
Pediatric Case Frequency Within the Past Year

The median range for respondents between consultation and radiotherapy start date was six to 10 days, although 9% noted averages >26 days. Regarding support, 50% had access to nutrition, 55% to rehabilitation, 57% to endocrinology, and 55% to anesthesia. Additionally, 14% had access to fertility services, 27% had access to neurocognitive support, 11% responded they had no support, and only one respondent had child life support. When asked what challenges they faced as physicians, 20% expressed a lack of treatment slots (meaning time available to treat a patient) with subsequent extended delay to new start, and 16% noted challenges with access to pediatric oncology and multidisciplinary teams.

Radiotherapy modality access and use was also queried for participants. Their responses are detailed in Table 2. The most common modality used to treat pediatric patients was intensity-modulated radiation therapy (IMRT).

**TABLE 2:**
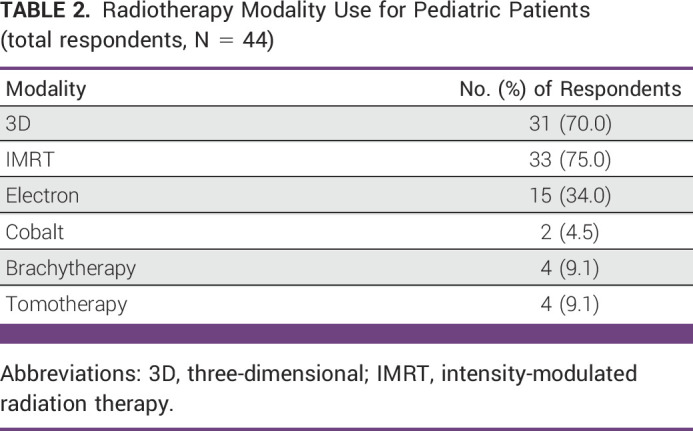
Radiotherapy Modality Use for Pediatric Patients (total respondents, N = 44)

### Aim Two: Educational Intervention

Through the SOMERA listserv, we successfully sent out case homework 2 weeks before the workshop on November 28, 2020. In total, 238 participants registered for the workshop. Author discussion of the three most common cases identified (Hodgkin lymphoma, medulloblastoma, and Wilms tumor) led to re-evaluation of Wilms tumor as a discussion case as it is still commonly treated with three-dimensional (3D) technology and thus well-understood in Mexico. With consensus, the team replaced Wilms tumor with the next most common answer, which was high-grade glioma.

**FIG 2:**
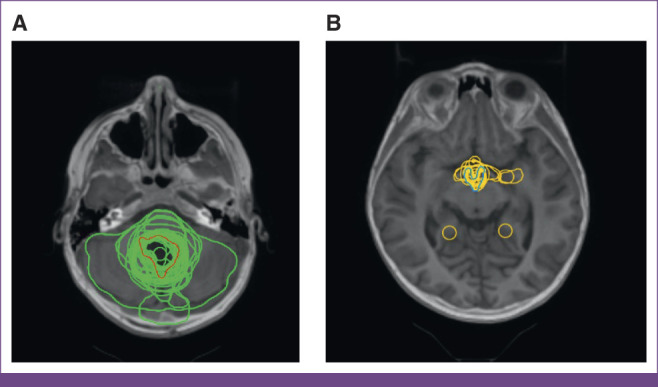
Representative contouring slices obtained before the workshop for a medulloblastoma case. (A) The appropriate target volume of the tumor bed (red) and the participant contours (green); (B) the hypothalamus for both reference (blue) and participant attempts (yellow).

A total of 238 participants registered for the workshop. Registration did not mandate homework completion. Forty-seven, 48, and 36 participants completed both preworkshop and posthomework for medulloblastoma, Hodgkin lymphoma, and high-grade glioma, respectively. Sample contour attempts from homework for medulloblastoma are shown in Figure 2. Dice metrics are displayed in Table 3. Significant improvement in Dice metrics was seen for all target volumes for all three cases. Regarding organs at risk (OARs), preworkshop mean Dice scores were highest for the optic chiasm, right optic nerve, and heart, and no significant improvement was seen for those structures. Significant improvement was seen in contours of the cochlea, hippocampal head, and hypothalamus.

**TABLE 3:**
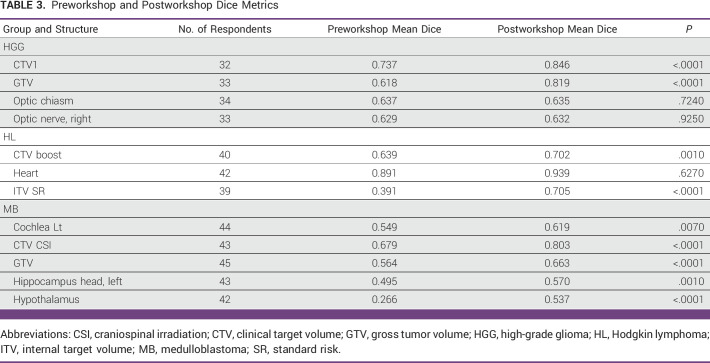
Preworkshop and Postworkshop Dice Metrics

## DISCUSSION

This work represents the culmination of two important specific aims. We present the first national survey of Mexican radiation oncologists to explore pediatric radiotherapy capacity. We also present the results of the first Latin American e-contouring workshop educational intervention with preworkshop and postworkshop Dice metrics. From the results of our educational intervention, we noted statistically significant improvement in all target volumes and many OARs. By and large, the success of this work reflects the collaborative nature between participants across national borders and the influential support of SOMERA. In tackling this effort, the predetermined greatest limitation for both aims was participation.^16^ With SOMERA's support and distribution, the survey was able to capture a wide and representative swath of Mexico, corresponding to the states with the highest population (Fig 1). That representation is depicted more granularly in Table 4. Similarly, it is with the support of SOMERA and their legitimization of these efforts through the offering of CME credits that we are able to present the first Latin American experience of e-contouring demonstrating efficacy through preintervention and post-intervention results. Alongside every step since the initial stakeholder meeting, the survey and workshop reflect the questions, ideas, and intents that Mexican investigators and decision makers felt important to include.

**TABLE 4:**
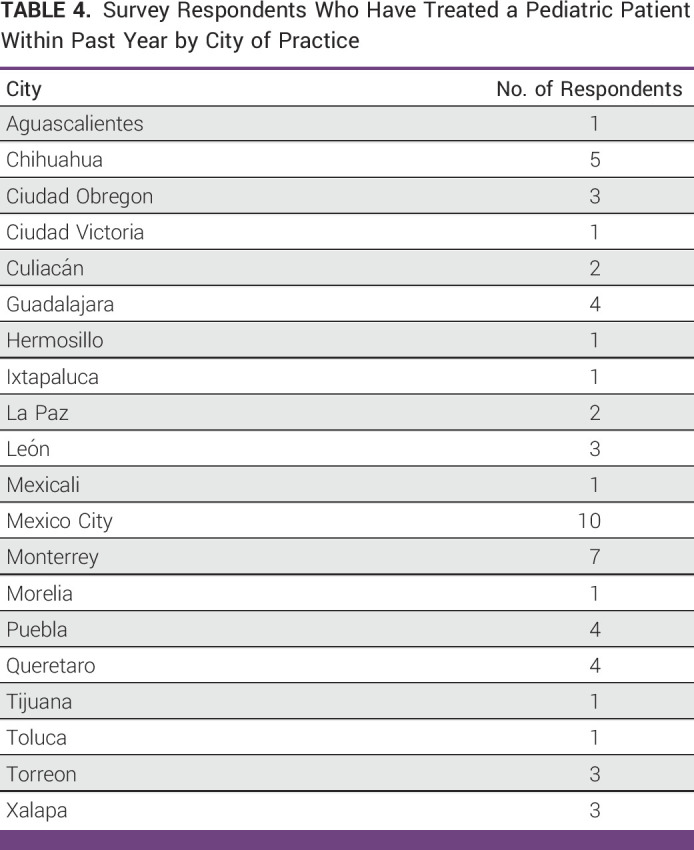
Survey Respondents Who Have Treated a Pediatric Patient Within Past Year by City of Practice

Children with cancer in Latin America face worse outcomes than do American children.^1^ Evaluations for these disparities in survival highlight access to care, resources, labor shortage, and pediatric-specific training.^4^ Mexico was selected as our pilot location for survey and educational intervention owing to its population size in Latin America and its higher incidence of childhood cancers compared with other Latin American nations. With our Mexican partners and pediatric thought leaders, we devised a survey to best understand the current radiation capacity for treating children and the challenges that radiation oncologists face. The management of pediatric tumors is complex and highly specialized. As such, it is common for parents to be inordinately burdened with relocation to institutions experienced in this training.^17^ Latin American countries such as Mexico face a shortage of pediatric-trained oncologists, and those centers are typically located in a few cities across the country. Notably, of the radiation oncologists who have treated children within the past year, approximately one of four said they did not feel comfortable treating pediatric patients. This finding was not significantly associated with patient volume, although such an analysis may be underpowered. Notably, the median volume reported of 9.5 patients does belie a treatment pattern that almost half of the respondents who see children see approximately fewer than one pediatric patient a month. These data reveal that most pediatric patients with cancer are seen by radiation oncologists with limited experience treating children or those for whom young patients are not a focus of their practice and training. With such a volume, it would be understandable that rare cases (such as pediatric ones) may be challenging and intimidating. In another vein, Mexico's uptake of 2D to 3D therapy to IMRT was rapid when compared with the time course of adoption in the United States^5^; such rapid implementation of a newer technology without associated contour-based training for rare and challenging pediatric cases may be a barrier to improving outcomes for children. This is of elevated concern given that 75% of our respondents reported use of and/or access to IMRT for their treatment of pediatric cases, a modality highly dependent on accurate contouring of both target volumes and OAR.

The landscape analysis provides Mexican shareholders with important information regarding the barriers and challenges that radiation oncologists face. For pediatric oncology, optimizing the quality of survivorship is nearly as important as the pursuit of cure. To that end, a majority of respondents affirmed access to nutrition, rehabilitation, endocrinology, and anesthesia. This is reassuring; however, access to fertility services, neurocognitive support, and child life specialists all represent major areas for growth. Unfortunately, approximately one of 10 respondents indicated that no support system was available. Similarly, when given an open-ended chance to reply with self-identified challenges, 20% noted that delays to new start secondary to radiation treatment slot availability as significant challenges, with about one in 10 reporting a month-long wait between consultation and new start. When considering time-sensitive pathologies requiring radiation where delay has been associated with inferior outcomes such as medulloblastoma and Wilms tumor,^18,19^ these barriers can translate to worse cancer control and subsequently survival. Similarly, the management of pediatric cancers is complex, and yet only one in four respondents noted access to a multidisciplinary tumor board: a challenge corroborated when respondents answered challenges they face. Multidisciplinary tumor boards in the United States have been associated with alterations in interpretation of clinical data in 35% of patients, with most of those changes resulting in altered treatment. Lack of communication with specialists on the care team in such a setting represents a targetable area for improvement.

For our second aim, we sought to demonstrate the efficacy of a contouring-based educational workshop. Past attempts in Latin America had been faced with poor completion of homework,^16^ which prohibited demonstration of efficacy. Of the 238 participants registered, approximately 20% completed preworkshop and postworkshop homework, which allowed us to evaluate the efficacy of the intervention. We noted significant improvement after the intervention for all target volumes. The legitimization of educational efforts through SOMERA's offering of CMEs represents a major change from past attempts with an obvious boon in participation. This success offers a new pathway for successful educational interventions as well as homework completion, which is needed to demonstrate efficacy.

Despite the successes, there are limitations to our analysis. This study represents the first survey evaluating pediatric radiotherapy capacity, and more investigation is required to determine all contributing factors and their interplay that may lead to published inferior outcomes for children. Similarly, although participation has increased owing to the support of Mexico's national radiation oncology organization, of the 238 registered, only 20% of participants completed the preworkshop and postworkshop homework. Although true that all would benefit from the didactic content presented, there is vast room for improvements in engagement. To expand programming (be it successful), efficacy must be demonstrated (and significant) which requires an adequate sample size.

In conclusion, the goal of the survey was to provide an analysis of the landscape of Mexico's radiotherapy capacity in the treatment of childhood cancers. We present those results and underscore the participant's self-defined challenges of delay in radiotherapy start and need for multidisciplinary tumor boards. We demonstrate the first reporting of a Spanish language e-contouring session in Latin America. For our educational intervention, we noted statistically significant improvement in all target volumes using the Dice metric. We report improved participation compared with previous efforts through SOMERA partnership and CME incentivization.

## Data Availability

The authors agree to share anonymized data upon reasonable request by researchers.
